# Right Upper Quadrant Pain Following Endoscopic Retrograde Cholangiopancreatography; a Case Report

**DOI:** 10.22037/aaem.v10i1.1535

**Published:** 2022-03-16

**Authors:** Lan Thi Nguyen, Dang Hai Do, An Duc Thai, Hoa Thi Nguyen

**Affiliations:** 1 Department of Hepatobiliary Surgery, VietDuc University Hospital, Hanoi, Viet Nam.; 2 Department of General Surgery, Hanoi Medical University, Hanoi, Viet Nam.; 3 Center of Anesthesia and Surgical Intensive Care, VietDuc University Hospital, Hanoi, Viet Nam.

**Keywords:** Pancreatitis, acute necrotizing, peritonitis, cholangiopancreatography, endoscopic retrograde, case reports

## Abstract

Endoscopic retrograde cholangiopancreatography (ERCP) is a standard for diagnosing and treating hepato-pancreatico-biliary (HPB) diseases in clinical settings. ERCP-related complications are relatively common, ranging from 4 to 30%. The most common one is acute pancreatitis. ERCP-related necrotizing pancreatitis accounts for 7.7% of ERCP-related pancreatitis cases. This complication may still be misdiagnosed, which might lead to inappropriate treatment with a worse prognosis. Here, we report a 34-year-old case with ERCP-related necrotizing pancreatitis who was successfully managed, but initially misdiagnosed with biliary peritonitis.

## 1. Introduction

Endoscopic retrograde cholangiopancreatography (ERCP) is a common tool for diagnosing and treating hepato-pancreatico-biliary diseases. Via direct vision of the bile duct, sphincterotomy and gallstone removal are conducted, and it has truly become the standard treatment for common bile duct (CBD) stone removal nowadays. ERCP-related complications are relatively common, ranging from 4 to 30% [1]. Most of these complications are not too serious and can be conservatively treated; however, the challenges lie in critical cases. The mortality rate was reported to be 0.5-1.5% and as high as 18% in duodenal injury [2, 3]. 

Pancreatitis, bleeding, perforation, and cholangitis are the most common complications. Though the methods for diagnosis and treatment have been established, in some sophisticated cases, a misdiagnosis could still occur, which might lead to inappropriate treatment with a worse prognosis. We report successful surgical management for a 34-year-old case with ERCP-related necrotizing pancreatitis, which was first misdiagnosed as biliary peritonitis.

## 2. Case presentation:

A 34-year-old woman came to the emergency department with the symptoms of right upper quadrant pain and a mild fever for the past two weeks. Her husband reported a history of choledocholithiasis six months ago without any intervention. Three days before hospitalization, the patient underwent sphincterotomy and stone removal via ERCP in a regional hospital. The next day, she had intense pain and a high temperature (39°C). A computed tomography (CT) scan was conducted, which showed a sign of intra-abdominal free fluid, air, and fat stranding around the descending part of the duodenum; pancreatic parenchyma was normal, and there was no sign of infection. Suspected of having an ERCP-related perforation, ERCP was conducted again, and a plastic stent was inserted in the biliary duct. Simultaneously, abdominal drainage was also placed. However, her condition did not improve, and she was transferred to our hospital.

**Figure 1:**
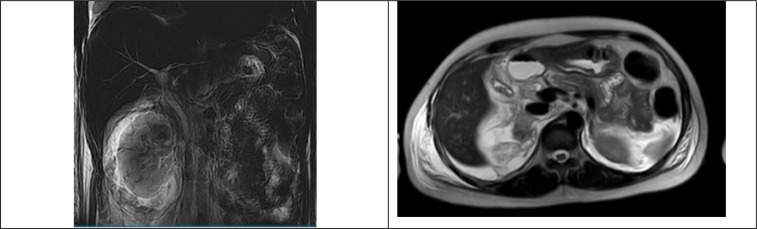
Coronal (left) and axial (right) views of abdominal Magnetic resonance imaging (MRI) at the time of admission

The patient was admitted to our hospital in an unstable state, with tachycardia and 38.5°C fever. The examination showed abdominal distension and right-sided abdominal rebound tenderness and ascitic fluid was spotted. Her blood test showed a high white blood cell count 13.2 G/l (98.7% Neutrophil), calcium 1.43 mmol/l, elevated alanine aminotransferase and aspartate aminotransferase levels (69 and 225.1 U/l, respectively), and total bilirubin 13 umol/l, amylase 349.9 U/L, and lipase 338.5 U/L. Bedside Index for Severity in Acute Pancreatitis (BISAP) score was 3 points, CT Severity Index (CTSI) score was 0 points. The magnetic resonance imaging (MRI) scan also suggested common bile duct perforation, with no clear sign of edematous or necrotizing pancreatitis (figure 1). An emergency operation was conducted 8 hours after admission. 

**Figure 2:**
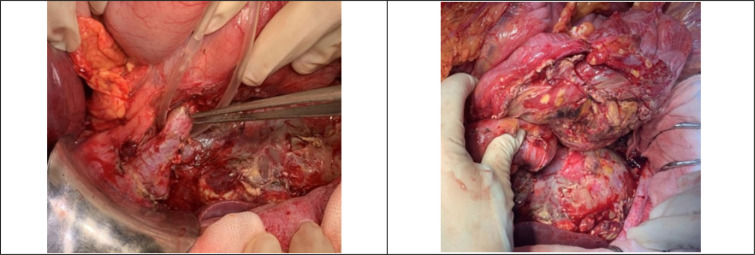
The operative findings of the reported case. The non-perforated common bile duct (left) and peripancreatic fat necrosis caused by necrotizing pancreatitis (right)

Unexpectedly, the cause was not an ERCP-related perforation (figure 2). Intraoperative findings revealed peripancreatic fat necrosis throughout the abdomen, severe inflammation in the head of the pancreas, and small residual common bile duct (CBD) stones (figure 2). She underwent CBD clearance and stone removal with T-tube drainage, cholecystectomy, and Witzel jejunostomy for feeding. Four drainage tubes were placed in the transhepatic, peripancreatic, and Doughlas regions. The calculated Imrie score and Acute Physiology and Chronic Health Evaluation (APACHE) II scores were 2 and 9 points, respectively, which means the patient was under severe conditions with the risk of mortality of 11-18%. 

She was immediately admitted to the intensive care unit and was managed critically with analgesia, antibiotics, somatostatin, and proton pump inhibitors for 10 days. Parenteral nutrition was applied and gradually turned into enteral nutrition via Witzel jejunostomy. Fortunately, the symptoms were relieved, and she was discharged 1 month later and was stable during the follow-up.

## 3. Discussion:

The number of ERCP procedures has been increasing in recent years, and ERCP is becoming a practical tool for clinicians in diagnosing and treating HPB diseases [4, 5]. Though the risk of complications is as low as 4%, severe complications are truly a matter of concern. 10-20% of them are classified as “severe” with an overall mortality rate of 1-1.5% [6, 7]. The most common complications include severe pancreatitis, perforation, and hemorrhagic shock. The overall risk increases in patients with sphincter of Oddi dysfunction and sphincterotomy, while severe complications are associated with systemic disease, obesity, prolonged procedures[6].

ERCP-related necrotizing pancreatitis is a rare condition, accounting for 7.7% of ERCP-related pancreatitis [8]. In a study by Fung on 72 patients with necrotizing pancreatitis, 6 of the cases (8.3%) were caused by ERCP, and they also had a higher APACHE score [9]. Potential mechanisms may originate from mechanical, chemical, thermal, hydrostatic, biochemical factors. More specifically, papillary edema and spasms cause pancreatic fluid obstruction and pancreatitis. Besides, activated pancreatic enzymes may damage and “autodigest” the parenchyma, while other factors such as injection pressure, iatrogenic, contrast media, and thermal injury may facilitate the process [10]. 

A retrospective study by Vege showed that 7.7% of severe acute pancreatitis cases were caused by ERCP, with a mortality rate of 25%, slightly higher than other causes [8]. We also thought that a prognostic model should be adopted. However, since the patient had high APACHE II and Systemic inflammatory response syndrome (SIRS)24-48 scores but low SIRS0-24 score diagnosing her problem was really challenging [8, 11].

At first, we misdiagnosed the patient with ERCP-related bile duct perforation. Examination showed right-sided rebound abdominal tenderness while the previous ERCP showed thickened, inflamed papilla. MRI also suggested a 3 mm perforated site in the lower part of the common bile duct, free fluid with no air in the peripancreatic and retroperitoneal region. Emergency operation was chosen as previous evidence showed their benefits for patients with unstable conditions [12, 13]. The symptoms were then determined to be caused by pancreatic necrosis and no sign of perforation was spotted. 

Interestingly, a research by Fathi on 2447 patients, with 6.9% complications, also showed that rebound tenderness did not occur in pancreatitis, but is suggestive of perforation [7]. While the two conditions had different approaches, we thought that severe pancreatitis should not be excluded when peritonitis is present and prophylaxis for high-risk patients should be further studied. In this case, ERCP and MRI could not be a gold standard since the papilla was inflamed. If a patient’s conditions do not improve, the doctor should promptly head to another morbidity. And finally, Witzel jejunostomy should be applied since it modulates the inflammatory response and reduces the rate of organ failure better than parenteral nutrition.

## 4. Conclusion:

ERCP-related necrotizing pancreatitis is a truly hazardous condition and might be misdiagnosed with other causes of peritonitis. A comprehensive study should be conducted, and larger studies are needed to find the best approach for its management.

## 5. Declarations:

### 5.1 Acknowledgement

We would like to express our thanks to all the medical staff in Department of Hepatobiliary surgery, Center of Anesthesia and Surgical intensive care, Medical Imaging and Nuclear Medicine Center ,for their contribution to diagnosis and management of the patient. 

### 5.2. Source of Support and Funding

None.

### 5.3. Conflict of Interest

None.

### 5.4. Ethical statements

Written informed consent was obtained from the patient for publication of this case report and accompanying images. No identity will be published, and all the published information will be used for education purposes only. The patient understands that name and initials will not be published, and due efforts will be made to conceal identity, but anonymity cannot be guaranteed. The study was approved by our institutional research committee.

### 5.4. Author contribution

Lan NT: Main surgeon, wrote manuscript, did the management strategy

Dang DH: Assistant surgeon, wrote manuscript, did the management strategy

An NT: Assistant surgeon, wrote manuscript

Hoa NT: Did the anesthesia, data collection
